# Supplementary material for: Regulating Au coverage for the direct oxidation of methane to methanol
Source: Nat Commun. 2024 Jan 17;15:564. doi: 10.1038/s41467-024-44839-6 (PMC10794185; doi:10.1038/s41467-024-44839-6)
Supplement: Supplementary file 1 — Supplementary Information [file 41467_2024_44839_MOESM1_ESM.pdf]

Supplementary Information

Regulating the Au coverage for the direct oxidation of methane to methanol

Yueshan Xu[1,#], Daoxiong Wu[1,#], Qinghua Zhang[2,#], Peng Rao[1], Peilin Deng[1,*], Mangen Tang[1], Jing Li[1], Yingjie Hua[3], Chongtai Wang[3], Shengkui Zhong[4], Chunman Jia[1], Zhongxin Liu[1], Yijun Shen[1], Lin Gu[2,5,*], Xinlong Tian[1,*] and Quanbing Liu[6,*]

[1] School of Marine Science and Engineering, Hainan Provincial Key Lab of Fine Chemistry, School of Chemistry and Chemical Engineering, Hainan University, Haikou, 570228, China

[2]Beijing National Laboratory for Condensed Matter Physics, Institute of Physics, Chinese Academy of Sciences, Beijing, 100190, China

[3] Key Laboratory of Electrochemical Energy Storage and Energy Conversion of Hainan Province, School of Chemistry and Chemical Engineering, Hainan Normal University, Haikou 571158, China

[4]College of Marine Science & Technology, Hainan Tropical Ocean University, Sanya, 572022, China

[5]School of Materials Science and Engineering, Tsinghua University, Beijing, 100084, China

[6]Guangzhou Key Laboratory of Clean Transportation Energy Chemistry, Guangdong Provincial Key Laboratory of Plant Resources Biorefinery, School of Chemical Engineering and Light Industry, Guangdong University of Technology, Guangzhou 510006, China

[#] Y. Xu, D. Wu, and Q. Zhang contributed equally to this work.

***Corresponding authors**:*

dengpeilin@hainanu.edu.cn (P. Deng); lingu@mail.tsinghua.edu.cn (L. Gu);

tianxl@hainanu.edu.cn (X. Tian); Liuqb@gdut.edu.cn (Q. Liu)

TABLE OF CONTENT

Figures……………………………………………………………………………………………………3

Tables……………………………………………………………………………………………………35

Supplementary References……………………………………………………………………………...50

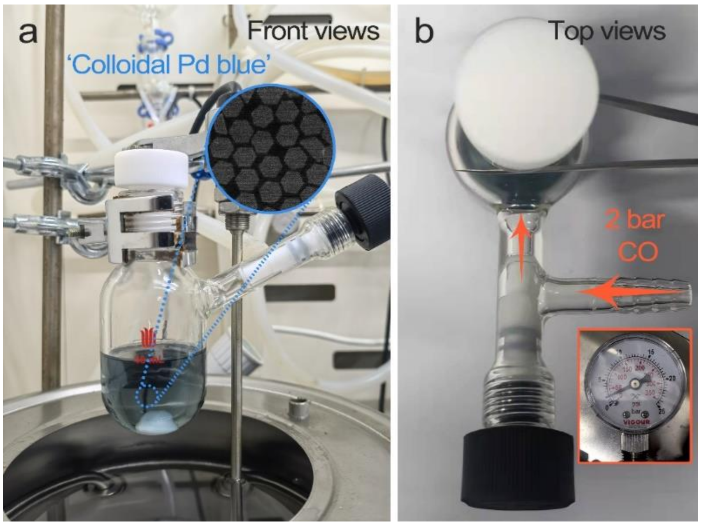

Supplementary Fig. 1 (a) Diagram of the Pd NS reaction equipment: 'Colloidal Pd blue'. (b) Airflow and pressure of CO.

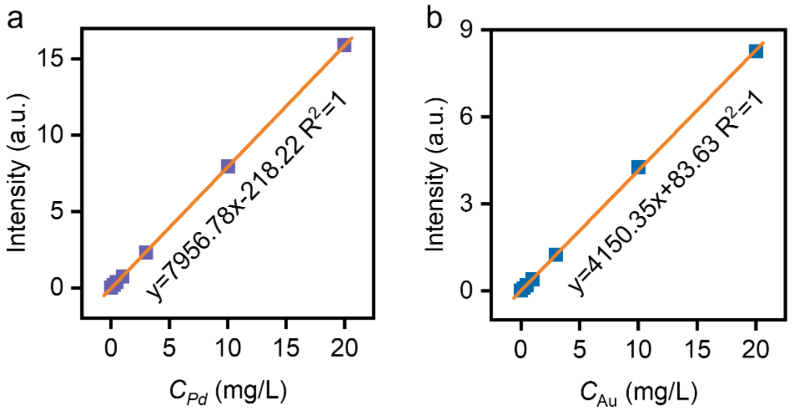

Supplementary Fig. 2 Standard curves of (a) Pd and (b) Au concentration with the corresponding signal values of the ICP-OES.

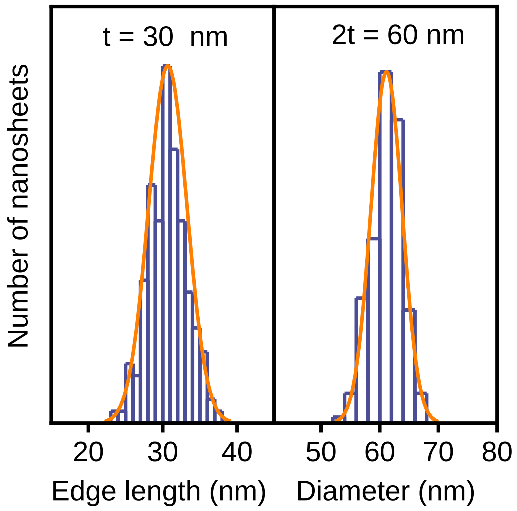

Supplementary Fig. 3 Edge length and diameter distribution of the Pd NS.

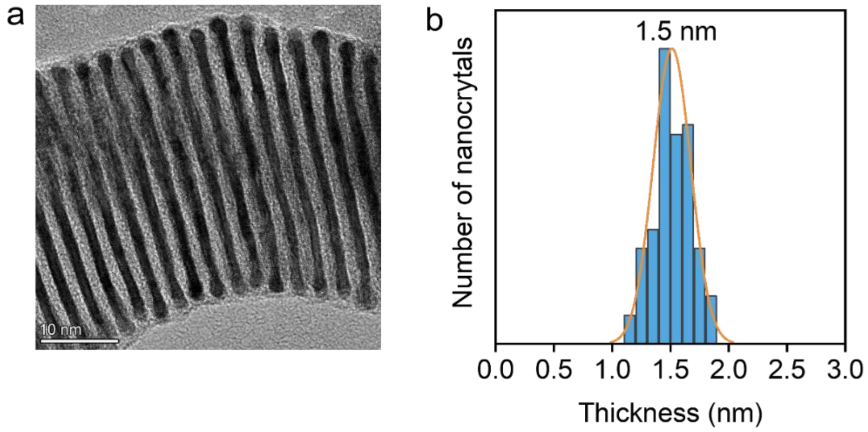

Supplementary Fig. 4 (a) Representative TEM of stacked Pd NS and (b) the thickness distribution of Pd NS.

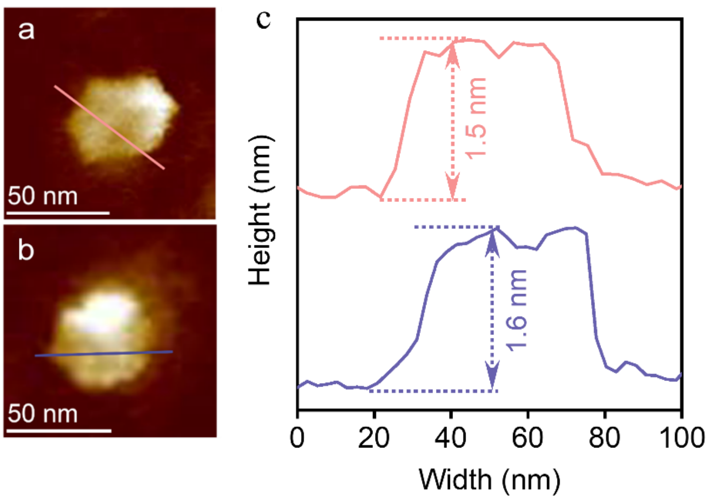

Supplementary Fig. 5 Atomic force microscope images of (a) Pd and (b) Pd_3Au_1 NS and (c) corresponding height profiles.

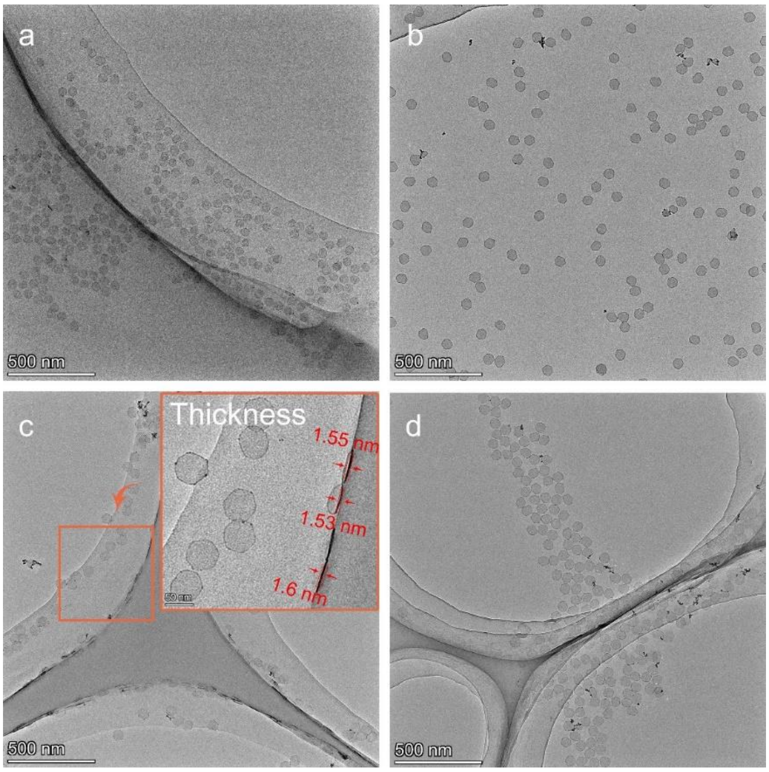

Supplementary Fig. 6 TEM images of (a) $Pd_{33}Au_1$ NS, (b) Pd_6Au_1 NS, (c) Pd_3Au_1 NS and (d) Pd_1Au_1 NS.

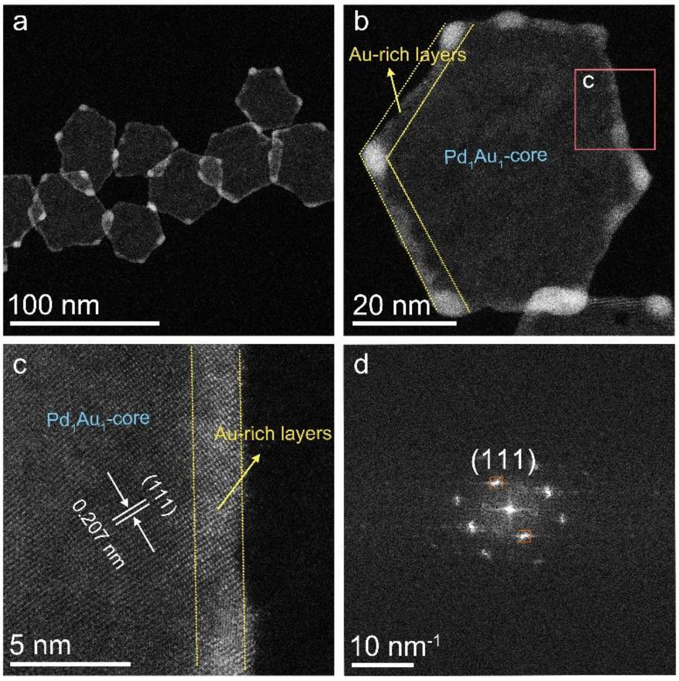

Supplementary Fig. 7 (a) Low magnification TEM image of Pd_1Au_1 NS, (b) TEM images of single Pd_1Au_1 NS and corresponding HRTEM images (c) and FTT pattern (d).

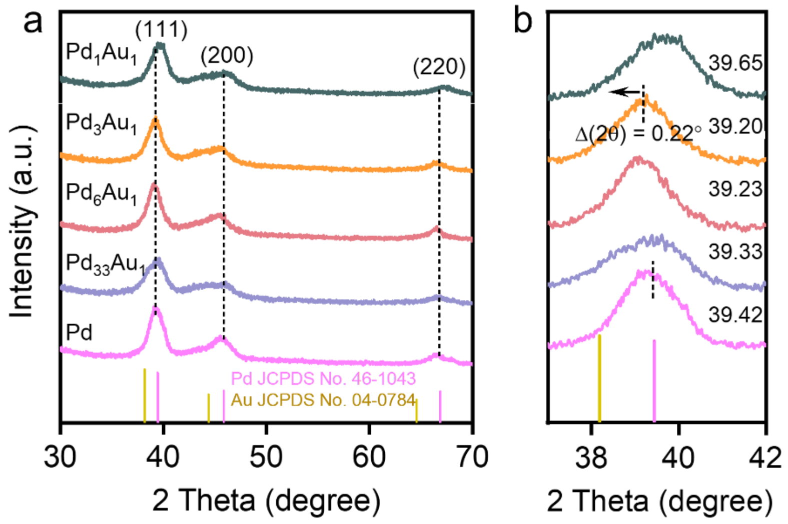

Supplementary Fig. 8 (a) XRD patterns of Pd and Pd_xAu_y NS. (b) The magnified image of the region around the crystal face of (111).

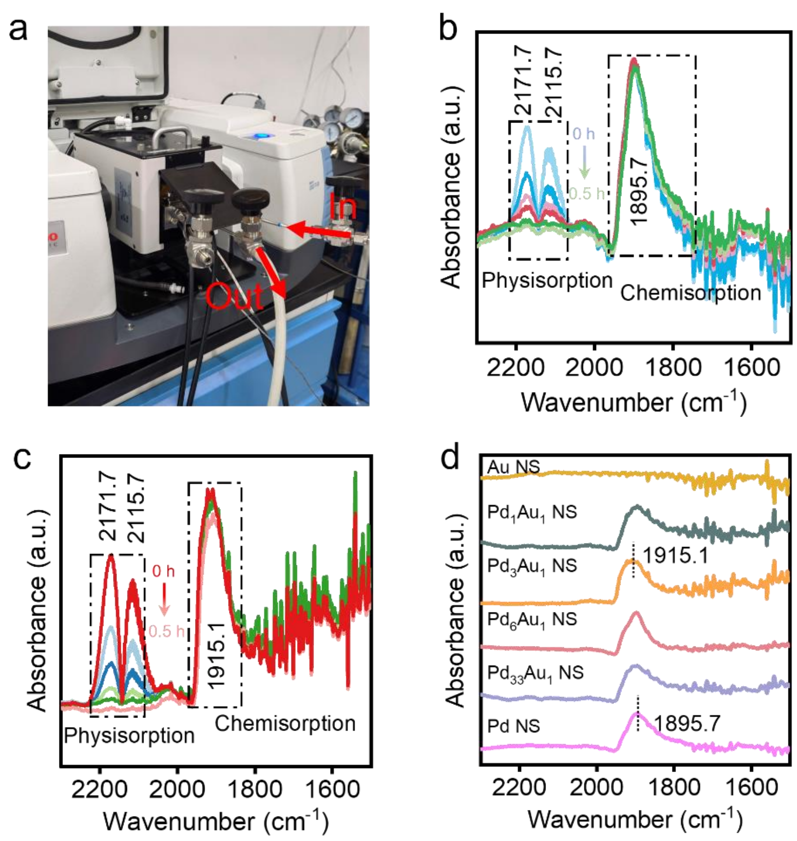

Supplementary Fig. 9 (a) The test device photograph of in-situ CO-DRIFTS. The in-situ CO-DRIFTS spectra of (b) Pd NS and (c) Pd_3Au_1 NS with the increasing time. (d) The in-situ CO-DRIFTS spectra of Pd NS, Au NS, and Pd_xAu_y NS.

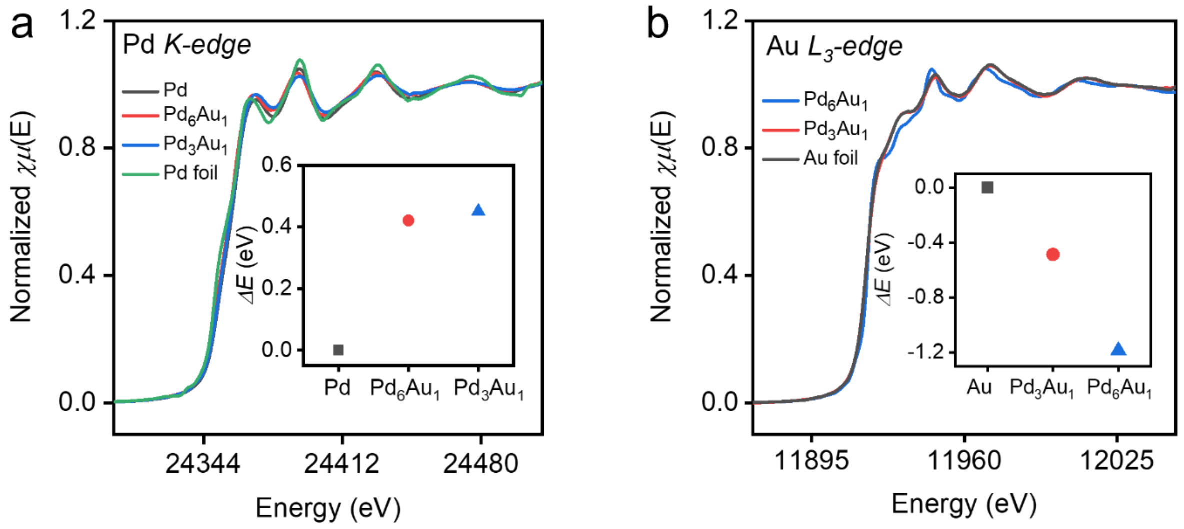

Supplementary Fig. 10 Fitting results of EXAFS characterization of the catalysts. (a and b) XANES spectra of the Pd *K*-edge and Au L_3-edge of Pd_xAu_y NS.

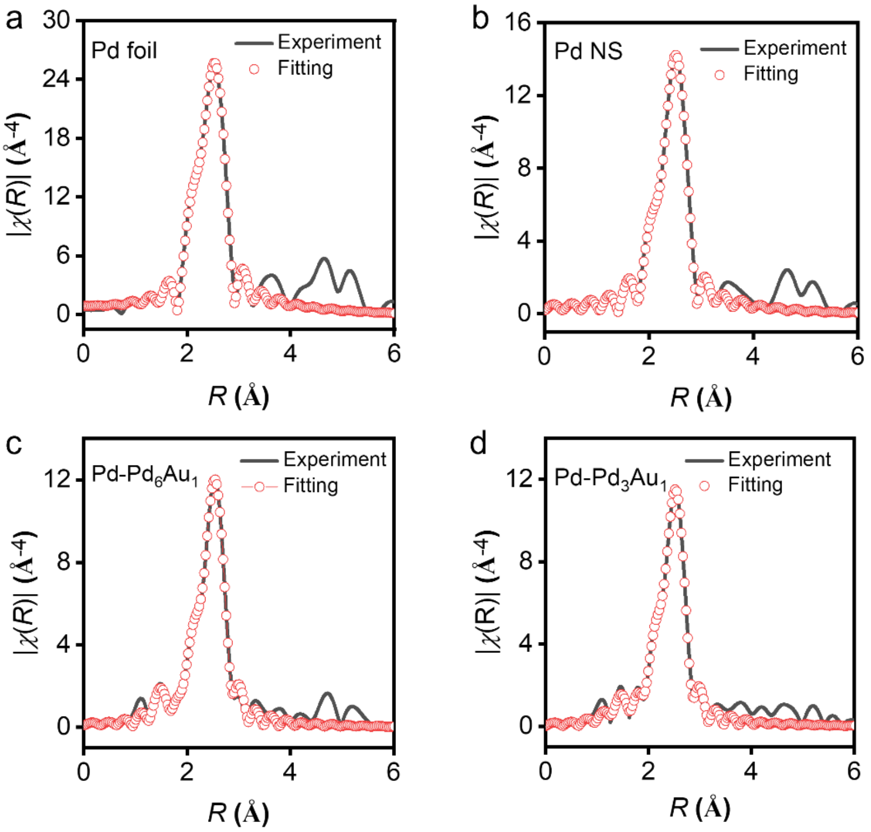

Supplementary Fig. 11 The fitting curve of EXAFS spectra of (a) Pd foil, (b) Pd NS, (c) Pd-Pd_6Au_1 NS, (d) Pd-Pd_3Au_1 NS.

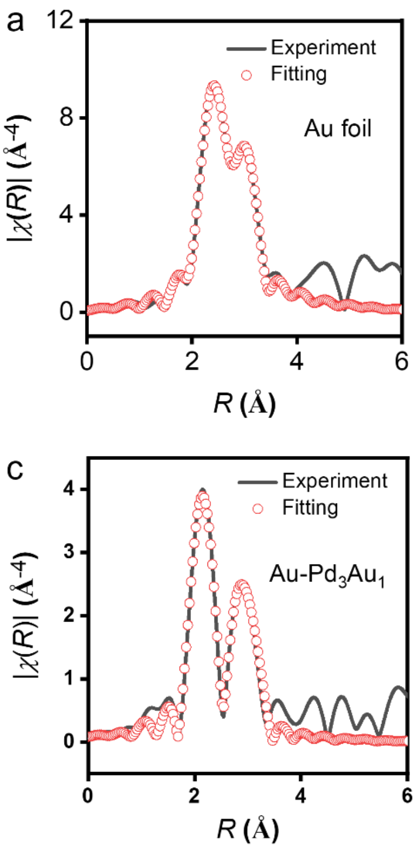

Supplementary Fig. 12 The fitting curve of EXAFS spectra of (a) Au foil, (b) Au-Pd_6Au_1 NS, (c) Au-Pd_3Au_1 NS.

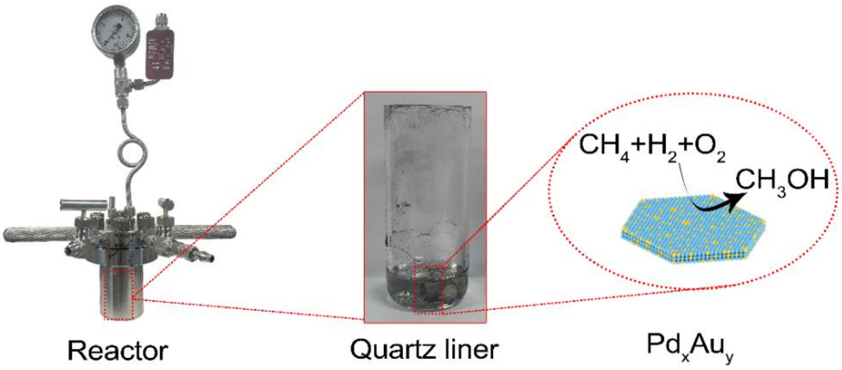

Supplementary Fig. 13 Scheme for the synthesis of CH_3OH from CH_4, H_2 and O_2 in a reactor.

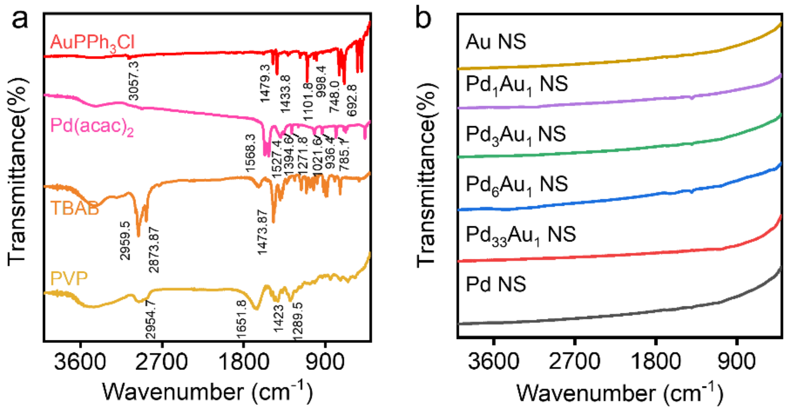

Supplementary Fig. 14 FTIR spectra of (a) four reactants (b) the obtained Pd, Pd_xAu_y, and Au NS.

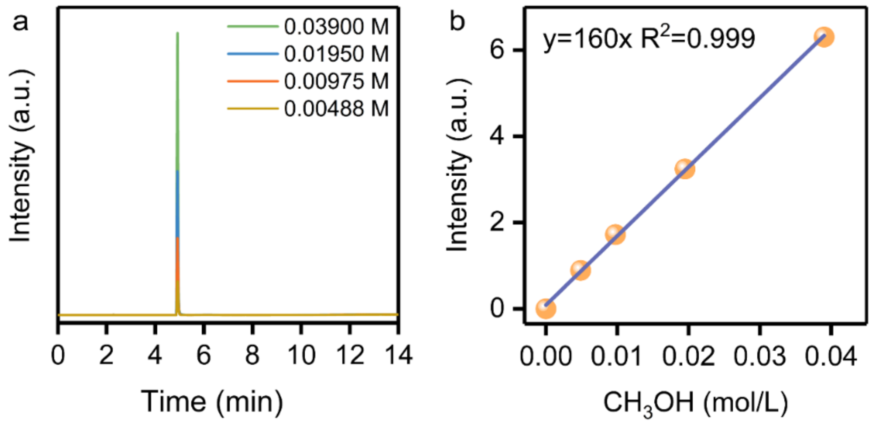

Supplementary Fig. 15 (a) The gas chromatograms signal of CH_3OH product with different concentrations; (b) The corresponding standard curves of CH_3OH.

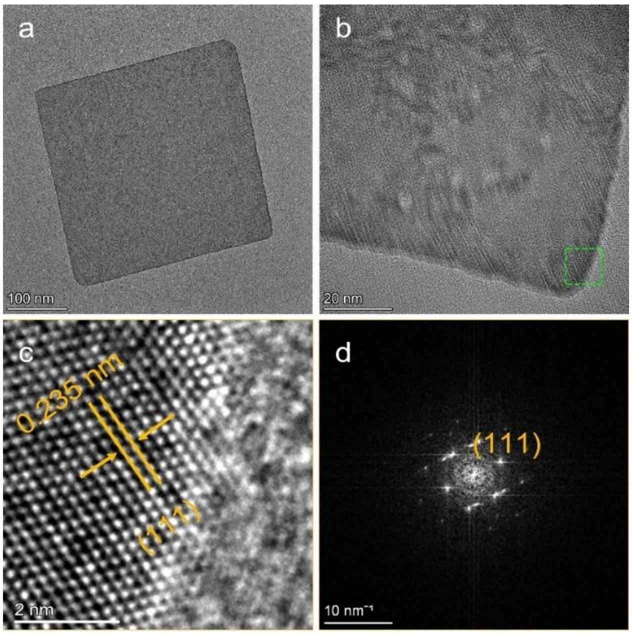

Supplementary Fig. 16 TEM image (a), high-magnification image (b-c) and corresponding FFT patterns (d) of Au NS.

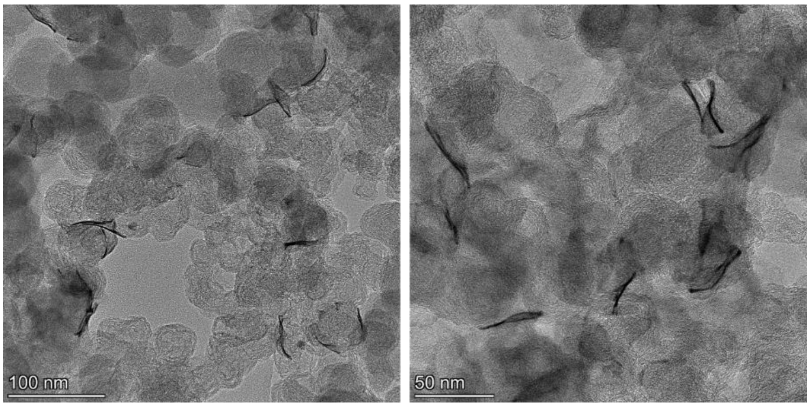

Supplementary Fig. 17 TEM images of Pd_3Au_1 NS/C Ns after the direct CH_4 oxidation test.

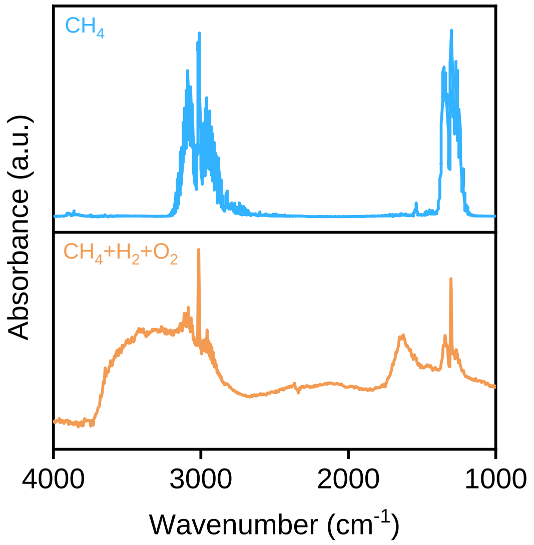

Supplementary Fig. 18 In situ DRIFTS spectra of adsorbed CH_4 and $CH_4/H_2/O_2$, respectively.

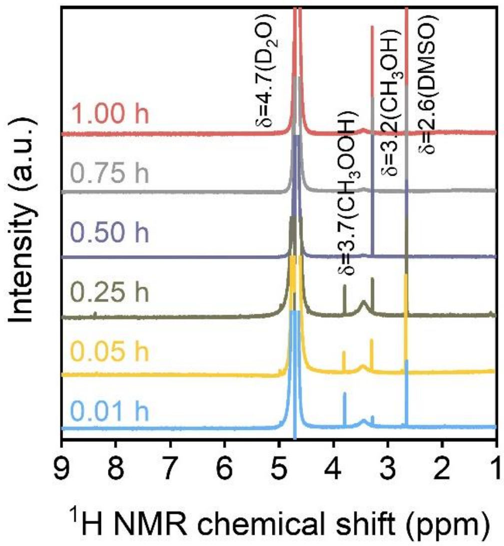

Supplementary Fig. 19 ^{1}H NMR spectra of liquid products with the reaction time from 0.01 h to 1 h.

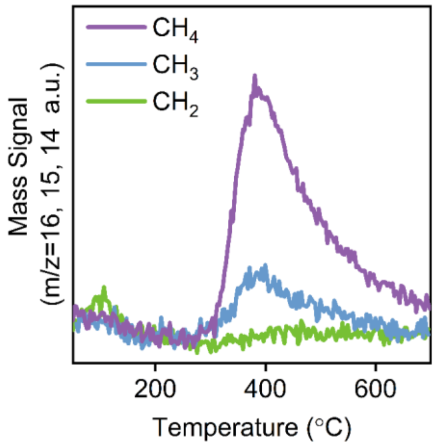

Supplementary Fig. 20 CH_4-TPD-MS results of Pd_3Au_1 NS.

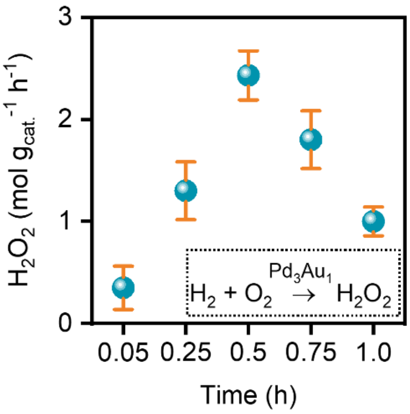

Supplementary Fig. 21 The time-dependency of in situ generation H_2O_2 productivity for 70 °C in O_2 and H_2 atmosphere.

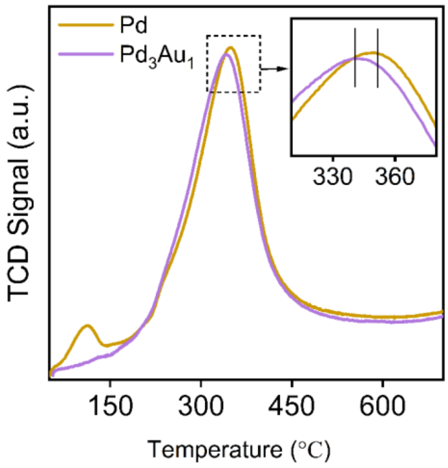

Supplementary Fig. 22 H_2-TPD of Pd_3Au_1 NS and Pd NS, respectively.

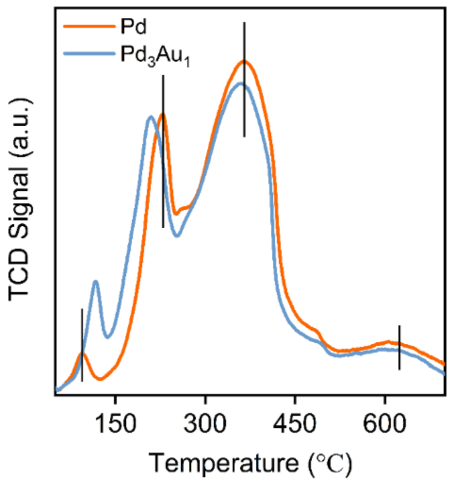

Supplementary Fig. 23 O_2-TPD of Pd_3Au_1 NS and Pd NS, respectively.

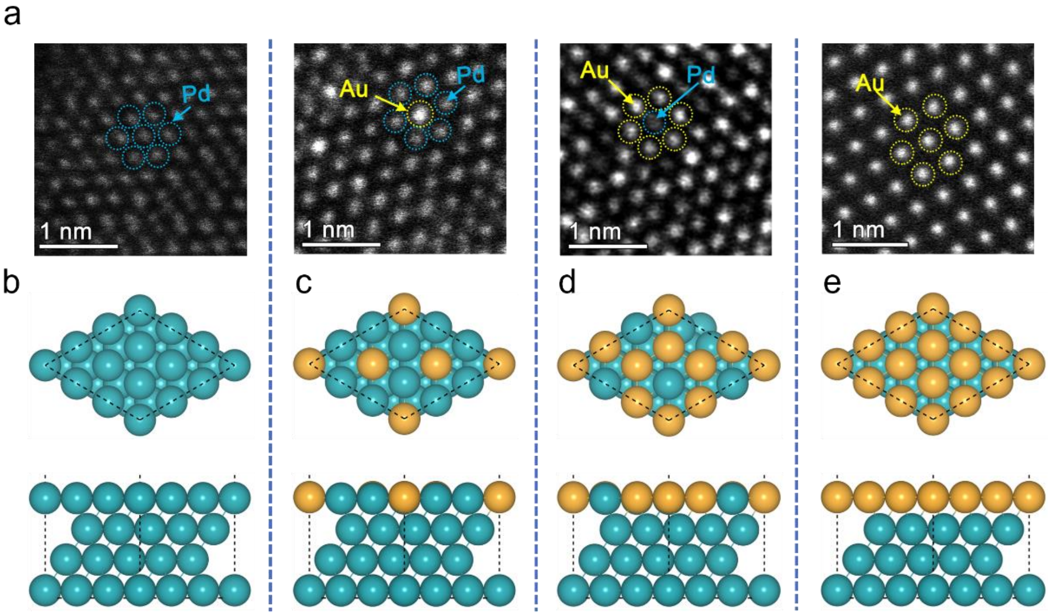

Supplementary Fig. 24 Atomic-resolution AC-STEM images of (a) Pd_xAu_y NS. Top view (upper) and side views (lower) of structural model for (b) Pd skin, (c) Pd_2Au_1 skin, (d) Pd_1Au_2 skin, and (e) Au skin.

In the prepared Pd_xAu_y NS samples, the distribution of Au and Pd atoms is not completely homogeneous. There are a variety of Au-Pd localized skin with different atomic arrangement configurations in the Pd_xAu_y NS samples, such as the pure Pd skin, the localized skin with one Au surrounded by Pd, the localized skin with one Pd surrounded by Au, and the pure Au skin, see Supplementary Fig. 23. Therefore, it is very difficult to reproduce the atomic structure of the prepared samples in one DFT model. Our DFT calculations aim to qualitatively investigate the behavior and microscopic mechanism of the influence of Au on the catalytic activity of Pd. Considering the computational consumption, the four representative models, including pure Pd skin, Pd_2Au_1 skin, Pd_1Au_2 skin, and pure Au skin, are selected in our calculations. Importantly, based on these four models, the DFT results are qualitatively consistent with the experimental results that the incorporation of a small amount of Au atoms into Pd nanosheets is beneficial for improving the performance of the DOMM. In addition, the DFT calculations show that the Pd_2Au_1 skin model exhibits the highest activity among the four models, which is a potentially active region in the catalyst. It is expected that the surface of the Pd_3Au_1 NS is dominated by the Pd_2Au_1 skin, resulting in the highest catalytic performance.

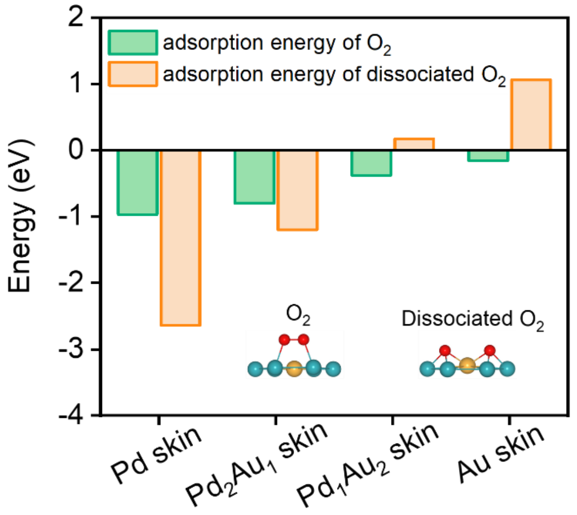

Supplementary Fig. 25 Adsorption energy (E_{ad}) of O_2 and dissociated O_2 on the surface. The adsorption energy is calculated according to $E_{ad} = E_{sysmtem} – E_{surface} – E_{adsorbent}$, where the E_{system}, $E_{surface}$, and $E_{adsorbent}$ are the DFT energy of the slab system with one O_2 or dissociated O_2 molecule adsorbed on the surface, the pristine surface, and one free-standing O_2 molecule. The difference between the adsorption energy ($E_{ad}(O_2) – E_{ad}(2O)$) of O_2 and dissociated O_2 are -1.67, -0.40, 0.55, and 1.22 for Pd skin, Pd_2Au_1 skin, Pd_1Au_2 skin, and Au skin, respectively.

Ham et al [1] showed that the efficient direct synthesis of H_2O_2 from H_2 and O_2 mediated by PdAu catalysts requires a moderate oxygen adsorption capacity on the catalyst surface. Our calculations show that the adsorbed oxygen molecules on the Pd skin and Pd_2Au_1 skin tend to dissociate into oxygen atoms ($E_{ad}(O_2) – E_{ad}(2O) < 0$ eV), while that on the Au skin is weak with ($E_{ad}(O_2) = -0.16$ eV). The adsorption of an O_2 molecule on Pd_1Au_2 skin is moderate ($E_{ad}(O_2) = -0.38$ eV) and its dissociation on the surface is thermodynamically unfavorable because the $E_{ad}(O_2) – E_{ad}(2O)$ value are positive. Thus, it is speculated that the Pd_1Au_2 moieties play an important role in the direct synthesis of H_2O_2 on the PdAu catalyst surface.

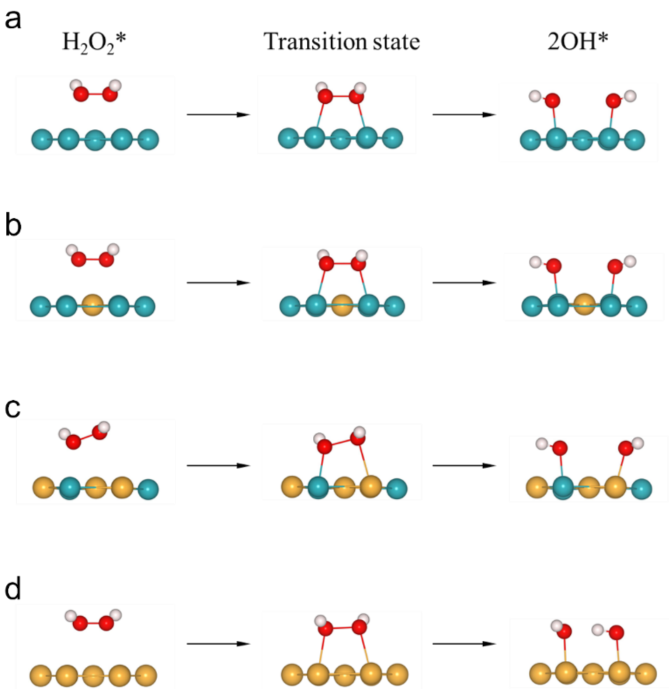

Supplementary Fig. 26 Reaction pathway of H_2O_2 decomposition into OH on the surface of (a) Pd skin, (b) Pd_2Au_1 skin, (c) Pd_1Au_2 skin, and (d) Au skin. For clarity, the bottom three layers of atoms are not shown.

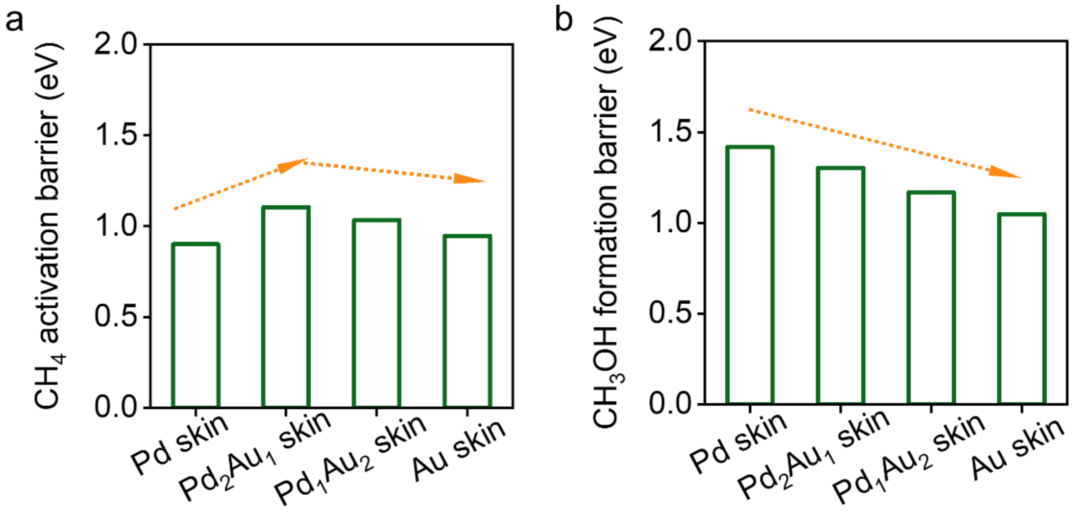

Supplementary Fig. 27 (a) CH_4 activation barrier and (b) CH_3OH formation barrier of Pd skin, Pd_2Au_1 skin, Pd_1Au_2 skin, and Au skin.

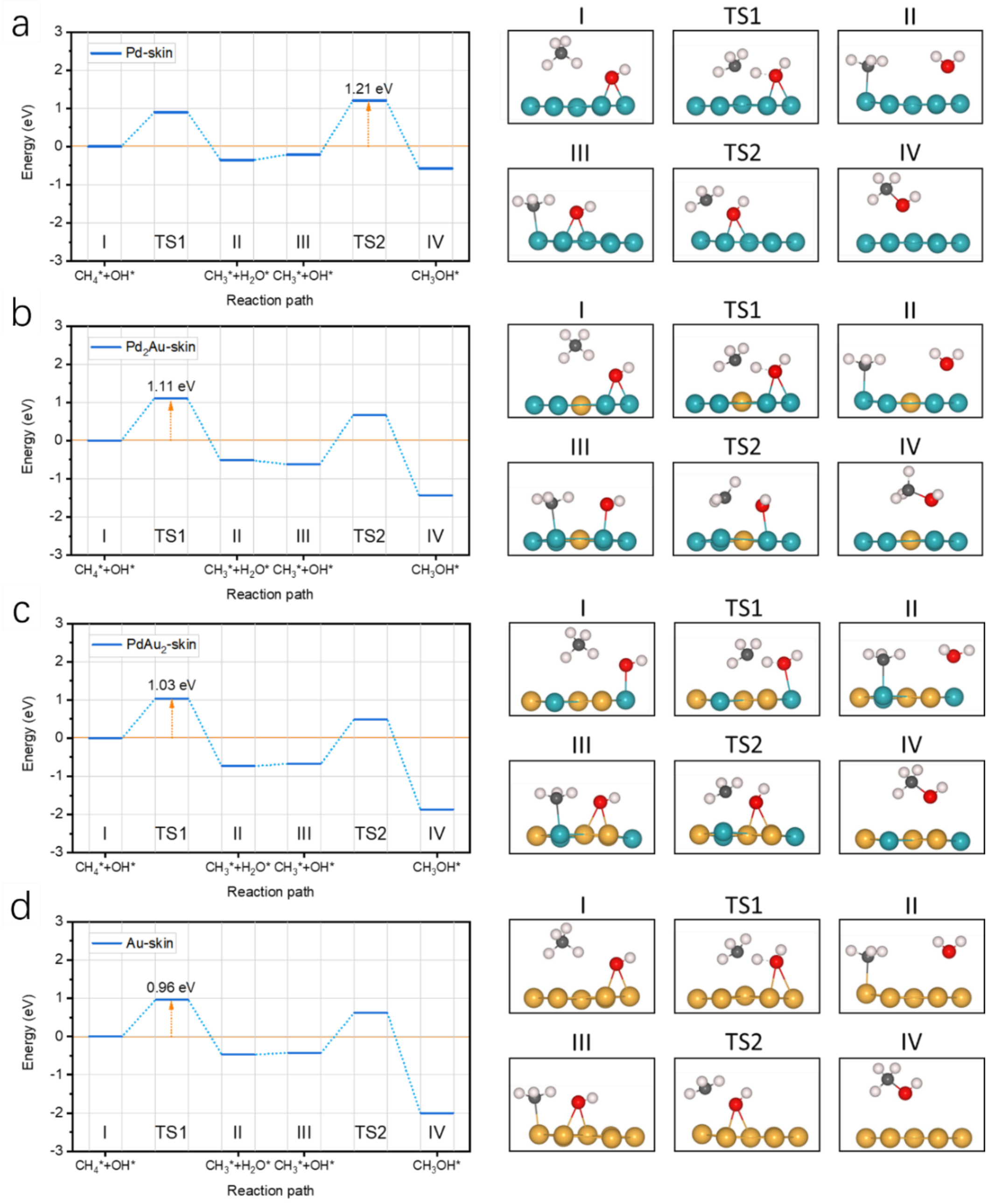

Supplementary Fig. 28 Reaction pathway of direct CH_4 oxidation to CH_3OH mediated by (a) Pd skin, (b) Pd_2Au_1 skin, (c) Pd_1Au_2 skin, and (d) Au skin. The apparent energy barriers are 1.21, 1.11, 1.03, and 0.96 eV for Pd skin, Pd_2Au_1 skin, Pd_1Au_2 skin and Au skin, respectively. For clarity, the bottom three layers of atoms are not shown. The data involved in reaction pathway of Pd skin and Pd_1Au_2 skin are taken from our previously published work[2].

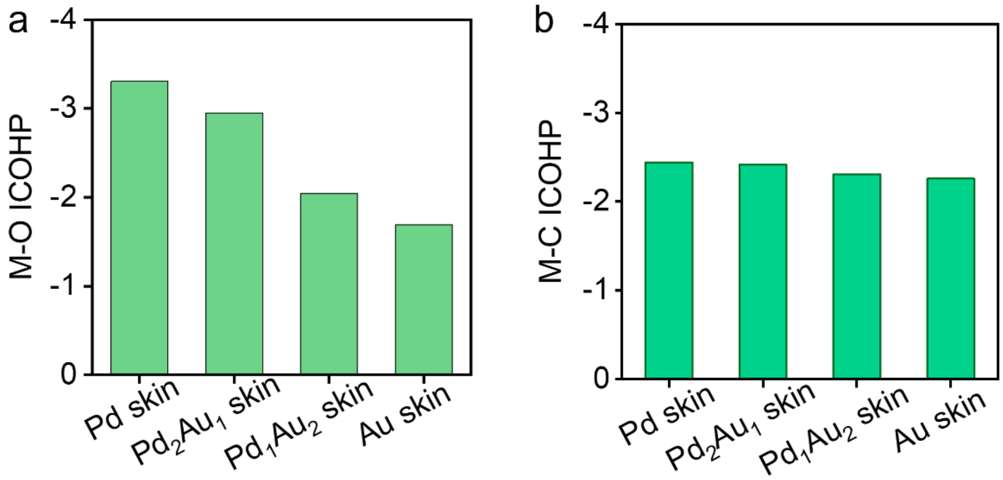

Supplementary Fig. 29 The value of (a) M-O ICOHP and (b) M-C ICOHP for Pd skin, Pd_2Au_1 skin, Pd_1Au_2 skin and Au skin. For Pd skin, Pd_2Au_1 skin and Pd_1Au_2 skin, M is Pd element, and for Au skin, M is Au element. The O and C denoted the oxygen atom in OH and carbon atom in CH_3 species adsorbed on the surface, respectively. The strength of M-O and M-C bond is evaluated by the Integrated Crystal Orbital Hamilton Population (ICOHP) at the Fermi level. A more negative ICOHP value corresponds to a stronger bond strength.

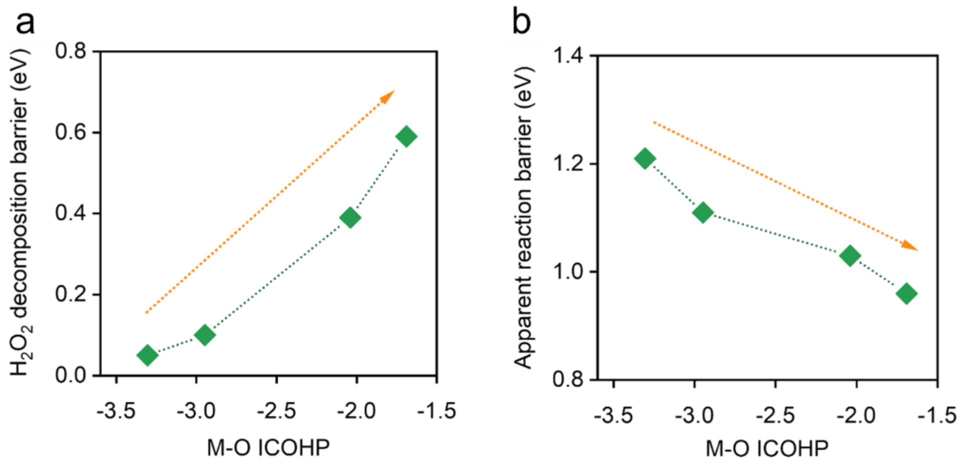

Supplementary Fig. 30 (a) H_2O_2 decomposition barrier and (b) apparent reaction barrier versus M-O ICOHP value. As the ICOHP value decreases, the H_2O_2 decomposition barriers are monotonically increasing from 0.05 to 0.59 eV and the apparent reaction barriers are monotonically decreasing from 1.21 to 0.96 eV.

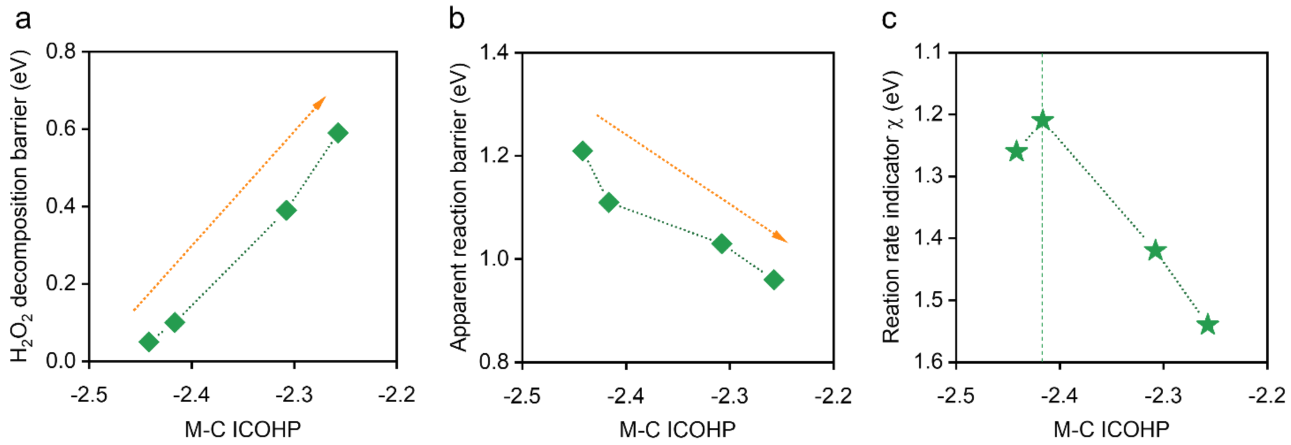

Supplementary Fig. 31 (a) H_2O_2 decomposition barrier, (b) apparent reaction barrier, and (c) reaction rate indicator c versus M-C ICOHP value.

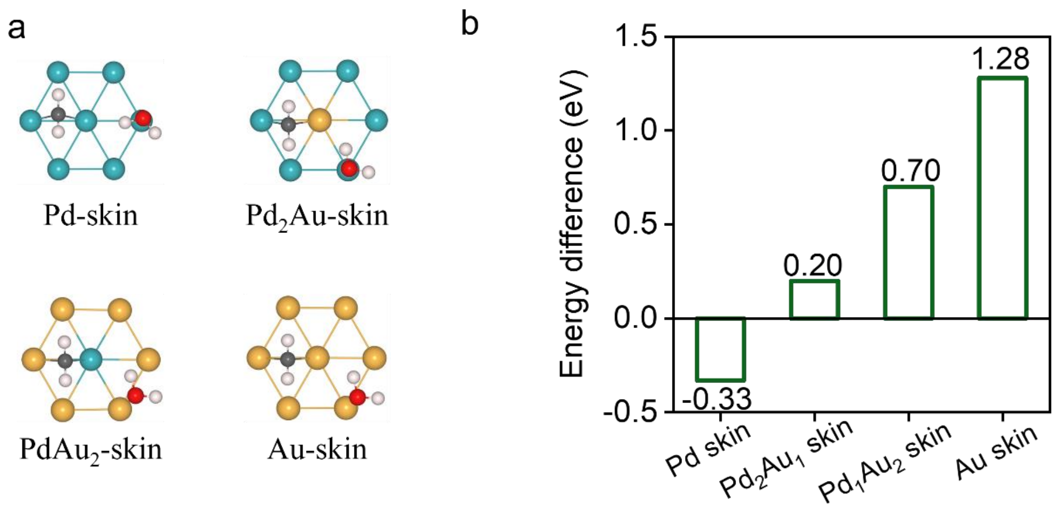

Supplementary Fig. 32 (a) Atomic structure of CH_2+H_2O configurations adsorbed on Pd_xAu_y NS surface. For clarity, the bottom three layers of atoms are not shown. (b) The energy difference between CH_3OH and CH_2+H_2O configurations. The positive energy difference of Pd_2Au_1 skin and Pd_1Au_2 skin indicate that the incorporation of Au atoms is beneficial for the inhibition effect of CH_3OH converted to HCOOH or CO_2 [3].

Supplementary Table 1. The detailed amounts of Pd NS seeds and $AuPPh_3Cl$ to prepare Pd_xAu_y NS with different Pd/Au molar ratios.

Catalysts	m_{AuPPh_3Cl} (mg)	n_{Au}(mol)	$V_{Pd\ NS}{}^{\xi}$(mL)	n_{Pd}(mol)	Pd/Au molar ratio
Pd_xAu_y NS	1.25	2.54×10^{-6}	5	9.4×10^{-5}	37:1
	7.50	1.52×10^{-5}			6.2:1
	12.50	2.54×10^{-5}			3.7:1
	25.00	5.08×10^{-5}			1.85:1

$^{\xi}$: 10 mg Pd NS contained in 5 ml DMF solution.

Supplementary Table 2. The Pd/Au atomic ratios of Pd_xAu_y NS from the ICP-AES measure.

Catalysts	m_0 (g)	V_0(mL)	Elements	C_0(mg/L)	Dilution factor: f	C_1(mg/L)$^{\psi}$	C_x(mg/kg)$^{\zeta}$	Pd/Au molar ratio$^{\Phi}$
$Pd_{33}Au_1$	0.0146	10	Au	0.60	100	60	41095.89	33:1
			Pd	10.77		1077	737575.34	
Pd_6Au_1	0.0297	10	Au	4.97	100	497	167426.60	6:1
			Pd	16.36		1636	550872.05	
Pd_3Au_1	0.0273	10	Au	5.61	100	561	205494.51	3:1
			Pd	9.19		919	336634.07	
Pd_1Au_1	0.0305	10	Au	17.31	100	1731	567868.85	1:1
			Pd	10.07		1007	330131.15	

m_0 is weighing by analytical balance.

V_0 is the volume of the fixed volume after dissolution.

C_0 is calculated by the external standard method.

ψ: $C_1 = C_0(mg/L) \times f.$

ζ: $C_x(mg/kg) = \frac{C_1(mg/L) \times V_0(mL) \times 10^{-3}}{m(g) \times 10^{-3}}$

Φ: $Pd/Au\ molar\ ratio = \frac{n_{Pd}(mol)}{n_{Au}(mol)} = \frac{C_{x,Pd}\left(\frac{g}{kg}\right) \times m_0(kg) \times 10^{-6}}{M\left(Pd, \frac{g}{mol}\right)} / \frac{C_{x,Au}\left(\frac{g}{kg}\right) \times m_0(kg) \times 10^{-6}}{M\left(Au, \frac{g}{mol}\right)}$

The C_0 represents the concentration of Pd or Au element in solution for Pd_xAu_y NS with unknown molar ratios of Pd/Au, which were obtained through the external standard method. Firstly, the standard curves were drawn with the known Pd or Au concentration and the corresponding signal values of the inductively coupled plasma optical emission spectroscopy (ICP-OES) (Supplementary Fig. 2). Next, the quantitative Pd_xAu_y NS dissolved in aqua regia of 10 mL was tested to obtain the signals values of Pd and Au elements by using ICP-OES. Finally, the C0 was calculated according to the standard curves and the signal values obtained by ICP-OES.

Supplementary Table 3. XPS spectra of Pd 3*d* peak for Pd NS and Pd_xAu_y NS.

Catalysts	B.E. (Pd $3d_{5/2}$)/eV	B.E. (Pd^{2+} $3d_{5/2}$)/eV	B.E. (Pd $3d_{3/2}$)/eV	B.E. (Pd^{2+} $3d_{3/2}$)/eV
Pd	335.92	336.90	341.18	342.66
$Pd_{33}Au_1$	335.99	337.33	341.25	342.91
Pd_6Au_1	336.15	337.71	341.43	342.99
Pd_3Au_1	336.23	337.88	341.49	343.19

Supplementary Table 4. XPS spectra of Au 4*f* peak for Au NS and Pd_xAu_y NS.

Catalysts	B.E. (Au $4f_{7/2}$) / eV	B.E. (Au $4f_{5/2}$)/eV
Au	84.34	88.01
Pd_3Au_1	83.95	87.58
Pd_6Au_1	83.92	87.56
$Pd_{33}Au_1$	83.88	87.52

Supplementary Table 5. Fitting results of the Pd *K*-edge and Au *L*-edge EXAFS date.

Entry	Catalysts	Edge	Shell	*CN*	*R* (Å)	ΔE_0(eV)	*R* factor
1	Pd	Pd *K*	Pd-Pd	10.12	2.78±0.01	4.5	0.001
2	Pd_6Au_1	Pd *K*	Pd-Pd	8.1±0.3	2.76±0.01	3.2	0.006
			Pd-Au	0.8±0.2	2.78±0.01		
		Au *L*	Au-Au	7.19±1.52	2.81±0.01	4.7	0.007
			Au-Pd	3.90±0.58	2.78±0.01		
3	Pd_3Au_1	Pd *K*	Pd-Pd	6.9±0.2	2.75±0.01	3.3	0.006
			Pd-Au	0.7±0.3	2.76±0.01		
		Au *L*	Au-Au	7.18 ± 1.57	2.82±0.01	4.1	0.012
			Au-Pd	2.59 ± 0.63	2.78±0.01		
4	Au foil	Au *L*	Au-Au	12	2.86±0.004	4.5	0.002

Supplementary Table 6. The catalytic performance of the direct CH_4 oxidation for Pd NS and Pd_xAu_y NS with different Pd/Au atomic ratios. The Pd_xAu_y NS supported on carbon blacks as catalyst for the direct oxidation of CH_4.

Entry	Catalysts	Amount of product (μmol)		Yield (mmol g^{-1} h^{-1})	Selectivity (%)	TOF/h^{-1}
		CH_3OH	CO_2			
1	Pd	3.64	0.15	72.8	96.0	92.2
2	$Pd_{33}Au_1$	4.87	0.13	97.5	97.4	139.8
3	Pd_6Au_1	5.66	0.13	113.1	97.8	218.4
4	Pd_3Au_1	7.39	0.15	147.8	98.0	404.5
5	Pd_1Au_1	4.75	0.14	95.0	97.1	685.4
6	Au	0.13	0.05	2.6	72.2	/

Supplementary Table 7. Experiment with blank contrast.

Entry	Reaction conditions	Amount of product (μmol)		Yield	Selectivity
		CH_3OH	CO_2	(mmol g^{-1} h^{-1})	(%)
1	3 MPa CH_4	0	0	0	0
2	3 MPa CH_4/10 uL H_2O_2	0.27	0.03	5.3	91.6
3	1.5 MPa CH_4/1.5 MPa O_2	0.13	0.02	2.5	85.0
4	1.5 MPa CH_4/ 1.5 MPa O_2/10 uL H_2O_2	0.17	0.02	3.5	88.3
5	1.5 MPa CH_4/1.5 MPa (O_2+H_2)	7.39	0.15	147.8	98.0
6	Carbon blacks 1.5 MPa CH_4/1.5 MPa (O_2+H_2)	0.09	0.01	1.8	90.0
7	Pd_3Au_1-TPP	1.88	0.07	37.6	96.4
8	Pd_3Au_1-SR_{12}	0.81	0.10	16.2	89.0

Entry 1-5: The Pd_3Au_1 NS supported on carbon blacks as catalyst for the direct oxidation of CH_4.

Entry 6-8: Carbon blanks, Pd_3Au_1-TPP and Pd_3Au_1-SR_{12} as catalyst for the direct oxidation of CH_4, respectively.

Supplementary Table 8. The turnover frequencies (TOF) of the Pd and Pd_xAu_y NS for DOMM.

Catalyst	n_{CH3OH} *(mol)*	$n_{surface}$ *(mol)*	δ *(%)*	$m_{cat.}$ *(g)*	*M (g/mol)*	*W (%)*	*T (h)*	TOF *(h^{-1})*
Pd	3.64×10^{-6}	9.4×10^{-7}	8.4					92.2
$Pd_{33}Au_1$	4.89×10^{-6}	8.9×10^{-7}	8.0					139.8
Pd_6Au_1	5.66×10^{-6}	7.2×10^{-7}	7.2	1×10^{-3}	106.42	10	0.5	218.4
Pd_3Au_1	7.39×10^{-6}	5.8×10^{-7}	6.3					404.5
Pd_1Au_1	4.75×10^{-6}	3.3×10^{-7}	4.2					685.4

Supplementary Table 9. The compared catalytic performance of various catalysts recently reported.

Entry	Catalyst	Conditions	CH_3OH Yield (mmol g_{cat}^{-1} h^{-1})	CH_3OH Selectivity (%)	Ref.
1	Pd_3Au_1 NS	3.0 MPa (0.0165 H_2/0.033 O_2/0.508 CH_4/0.3085 Ar/0.134 He), 25 °C, 0.5 h	43.7	95.0	**this work**
		3.0 MPa (0.0165 H_2/0.033 O_2/0.508 CH_4/0.3085 Ar/0.134 He), 70 °C, 0.5 h	147.8	98.0	
2	AuPd@ZSM-5-R	3.0 MPa 3.3% H_2/6.6% O_2/1.6% CH_4/61.7% Ar/26.8% He/70°C/0.5h	90.0	98.8	4
3	PdCu/ZSM-5	2.4MPaCH_4/0.8MPaH_2/0.3 MPa O_2	90	56	5
4	AuPd/TiO_2	30.5 bar CH_4 5000 µmol H_2O_2/70°C/0.5h	53.6	92	6
5	PMOF-RuFe(OH)	1 bar CH_4/O_2/3mL H_2O/20h	8.81	100	7
6	Fe-O Clusters	30 bar CH_4 300 µmol 30wt% H_2O_2	4.80	97.9	8
7	AuCu/ZnO	19 bar CH_4/1 bar O_2/100 mL H_2O/25 °C/2 h,	2.5	18.2	9
8	Cu_2@C_3N_4	1 MPa CH_4/0.5 MPa O_2/0.2 mL H_2O_2/50°C /0.5h	2.6	98	10
9	Rh/Pt/Ni-MMZ-IMP	2.0MPa CH_4/0.5MPa CO/0.2MPaO_2/150°C /3h	1.2	70	11
10	Rh-ZSM-5	20 bar CH_4/5bar CO 4 bar O_2/150°C/6h	1.22	8.78	12
11	AuPd/TiO_2	30.5 bar 0.86% H_2/1.72% O_2/75.86% CH_4/22.55% N_2/50°C/0.5h	0.14	89.2	13
12	Au-ZSM-5	20.7 bar CH_4, 1.0 bar O_2, 2.5 bar	0.136	74.1	14
13	Au_1/BP	33bar (CH_4:O_2 = 10:1) /90°C/2h	0.113	99%	15

14	ZnO/Fe_2O_3	1.0 bar CH_4/25°C/1.5h	0.12	100	16
15	Fe-BN/ZSM-5	10 bar 97%CO/N_2; 20 bar 95%CH_4/Ar; 654 μmol H_2O_2/50 °C/6h	0.08	20	17
16	Zr-UiO-66	5 bar CH_4 20 mL H_2O_2 50 °C/0.5h	0.025	7.1	18

Supplementary Table 10. The catalytic performance of the direct CH_4 oxidation with different reaction temperatures.

Entry	Temperature (°C)	Amount of product (μmol)			Yield (mmol g^{-1} h^{-1})	Selectivity (%)
		CH_3OH	CH_3OOH	CO_2		
1	25	2.18	0.005	0.11	43.7	95.0
2	50	4.40	0.00	0.13	88.0	97.1
3	70	7.39	0.00	0.15	147.8	98.0
4	90	4.89	0.00	0.25	97.8	95.1
5	110	3.21	0.00	0.35	64.2	90.2

Supplementary Table 11. The catalytic performance of direct CH_4 oxidation with different reaction time at 70 °C.

Entry	Time (h)	Amount of product (μmol)			Yield (mmol g^{-1})	Selectivity (%)
		CH_3OH	CH_3OOH	CO_2		
1	0.05	1.21	0.01	0.13	12.1	89.6
2	0.25	3.72	0.05	0.14	37.2	95.1
3	0.50	7.39	0	0.15	73.9	98.0
4	0.75	7.11	0	0.26	71.1	96.5
5	1.00	6.87	0	0.39	70.0	94.7

Supplementary Table 12. The catalytic performance of the direct CH_4 oxidation with different CH_4 % vol.

Entry	$P_{(CH_4)}/P_{(H_2+O_2)}$	Amount of product (μmol)		Yield (mmol g^{-1} h^{-1})	Selectivity (%)
		CH_3OH	CO_2		
1	0.02	1.44	0.19	28.8	87.9
2	0.50	3.62	0.33	72.3	91.6
3	1.00	7.39	0.15	147.8	98.0
4	2.00	3.93	0.36	78.7	91.7
5	5.00	1.09	0.19	21.8	85.1

Supplementary Table 13. The catalytic performance of H_2O_2 preparation from H_2 and O_2 for various catalysts recently reported. The Pd_3Au_1 supported on carbon blacks as catalyst for the direct oxidation of CH_4.

Entry	Catalyst	Conditions	Yield (mol g_{cat}^{-1} h^{-1})	H_2O_2 Selectivity (%)	Ref.
1	10% Pd_3Au_1 NS	1 mg/3.0MPa 0.0165H_2/0.033 O_2/0.508CH_4/ 0.3085 Ar/0.134 He/70°C/0.5h	2.4	~ 96%	**this work**
2	1%AuPd@ZSM-5-R	30 mg/3.0 MPa 3.3% H_2/6.6% O_2/1.6% CH_4/61.7% Ar/26.8% He/70°C/0.5h	0.12	~ 90%	4
3	2.5% Au–2.5% Pd/TiO_2	2.9 MPa 5% H_2/1.1 MPa25% O_2/70% CO_2/2°C/0.5h	0.11	~ 95%	19
4	3%Pd–2% Sn/TiO_2	2.9 MPa 5% H_2/1.1 MPa25% O_2/70% CO_2/2°C/0.5h	0.07	~ 96%	20
5	2.5% Au–2.5% Pd/C	10mg 2.9 MPa 5% H_2/1.1 MPa25% O_2/70% CO_2/2°C/0.5h	0.11	80 %	21

Supplementary Table 14. Summary of DFT results. The Underlined data are taken from our previously published work.[2]

	Pd skin	Pd_2Au_1 skin	Pd_1Au_2 skin	Au skin
H_2O_2 decomposition energy barrier (eV)	0.05	0.10	0.39	0.59
CH_4 activation energy barrier (eV)	<u>0.90</u>	1.11	<u>1.04</u>	0.96
CH_3OH formation barrier (eV)	<u>1.42</u>	1.28	<u>1.17</u>	1.05
Apparent reaction barrier (eV)	<u>1.21</u>	1.11	<u>1.03</u>	0.96
Reaction rate indicator χ (eV)	1.26	1.21	1.42	1.55
OH adsorption energy (eV)	-1.16	-0.86	-0.67	-0.31
Adsorption energy of O_2 (eV)	-0.97	-0.80	-0.38	-0.16
Adsorption energy of dissociated O_2 (eV)	-2.64	-1.20	0.17	1.06
M-O ICOHP	<u>-3.31</u>	-2.95	<u>-2.04</u>	-1.69
M-C ICOHP	<u>-2.44</u>	-2.42	<u>-2.31</u>	-2.26

Supplementary References

1. Ham, H. C.; Hwang, G. S.; Han, J.; Nam, S. W.; Lim, T. H., Geometric Parameter Effects on Ensemble Contributions to Catalysis: H_2O_2 Formation from H_2 and O_2 on AuPd Alloys. A First Principles Study. *J. Phys. Chem. C* **2010,** *114*, 14922-14928.
2. Xu, Y., et al., Au Decorated Pd Nanowires for Methane Oxidation to Liquid C1 Products. *Appl. Catal. B: Environ.* **2022,** *308*, 121223.
3. Zhu, K., et al., Highly Efficient Conversion of Methane to Formic Acid under Mild Conditions at ZSM-5-Confined Fe-Sites. *Nano Energy* **2021,** *82*, 105718.
4. Jin, Z., et al., Hydrophobic Zeolite Modification for in Situ Peroxide Formation in Methane Oxidation to methanol. *Science* **2020,** *367*, 193-196.
5. Wu, B., et al., Tandem Catalysis for Selective Oxidation of Methane to Oxygenates Using Oxygen over PdCu/Zeolite. *Angew. Chem., Int. Ed.* **2022,** *61*, e202204116.
6. Agarwal, N., et al., Aqueous Au-Pd Colloids Catalyze Selective CH_4 Oxidation to CH_3OH with O_2 under Mild Conditions. *Science* **2017,** *358*, 223-227.
7. An, B., et al., Direct photo-oxidation of methane to methanol over a mono-iron hydroxyl site. *Nat. Mater.* **2022,** *21*, 932-938.
8. Zhao, W., et al., Fe-O Clusters Anchored on Nodes of Metal-Organic Frameworks for Direct Methane Oxidation. *Angew. Chem., Int. Ed.* **2021,** *60*, 5811-5815.
9. Luo, L., et al., Binary Au–Cu Reaction Sites Decorated ZnO for Selective Methane Oxidation to C1 Oxygenates with Nearly 100% Selectivity at Room Temperature. *J. Am. Chem. Soc.* **2022,** *144*, 740-750.
10. Xie, P., et al., Oxo dicopper anchored on carbon nitride for selective oxidation of methane. *Nat. Commun.* **2022,** *13*, 1375.
11. Jia, X., et al., Library Creation of Ultrasmall Multi-metallic Nanoparticles Confined in Mesoporous MFI Zeolites. *Angew. Chem., Int. Ed.* **2021,** *60*, 14571-14577.

12. Shan, J.; Li, M.; Allard, L. F.; Lee, S.; Flytzani-Stephanopoulos, M., Mild oxidation of methane to methanol or acetic acid on supported isolated rhodium catalysts. *Nature* **2017,** *551*, 605-608.

13. Ab Rahim, M. H., et al., Oxidation of Methane to Methanol with Hydrogen Peroxide Using Supported Gold-Palladium Alloy Nanoparticles. *Angew. Chem., Int. Ed.* **2013,** *52*, 1280-1284.

14. Qi, G., et al., Au-ZSM-5 catalyses the selective oxidation of CH_4 to CH_3OH and CH_3COOH using O_2. *Nat. Catal.* **2022,** *5*, 45-54.

15. Luo, L., et al., Water Enables Mild Oxidation of Methane to Methanol on Gold Single-Atom Catalysts. *Nat. Commun.* **2021,** *12*, 1218.

16. Zheng, K., et al., Room-Temperature Photooxidation of CH_4 to CH_3OH with Nearly 100% Selectivity over Hetero-ZnO/Fe_2O_3 Porous Nanosheets. *J. Am. Chem. Soc.* **2022,** *144*, 12357-12366.

17. Wu, B., et al., Fe Binuclear Sites Convert Methane to Acetic Acid with Ultrahigh Selectivity. *Chem* **2022,** *8*, 1-15.

18. Fang, G., et al., Zirconium-oxo Nodes of MOFs with Tunable Electronic Properties Provide Effective · OH Species for Enhanced Methane Hydroxylation. *Angew. Chem., Int. Ed.* **2022,** *61*, e202205077.

19. Edwards, J. K., et al., Direct Synthesis of H_2O_2 from H_2 and O_2 over Gold, Palladium, and Gold-Palladium Catalysts Supported on Acid-Pretreated TiO_2. *Angew. Chem., Int. Ed.* **2009,** *48*, 8512-8515.

20. Freakley, S. J., et al., Palladium-Tin Catalysts for the Direct Synthesis of H_2O_2 with High Selectivity. *Science* **2016,** *351*, 965-968.

21. Edwards Jennifer, K., et al., Switching Off Hydrogen Peroxide Hydrogenation in the Direct Synthesis Process. *Science* **2009,** *323*, 1037-1041.
